# Dietary Intakes of Individual Flavanols and Flavonols Are Inversely Associated with Incident Type 2 Diabetes in European Populations^1^,^2^,^3^

**DOI:** 10.3945/jn.113.184945

**Published:** 2013-12-24

**Authors:** Raul Zamora-Ros, Nita G. Forouhi, Stephen J. Sharp, Carlos A. González, Brian Buijsse, Marcela Guevara, Yvonne T. van der Schouw, Pilar Amiano, Heiner Boeing, Lea Bredsdorff, Guy Fagherazzi, Edith J. Feskens, Paul W. Franks, Sara Grioni, Verena Katzke, Timothy J. Key, Kay-Tee Khaw, Tilman Kühn, Giovanna Masala, Amalia Mattiello, Esther Molina-Montes, Peter M. Nilsson, Kim Overvad, Florence Perquier, M. Luisa Redondo, Fulvio Ricceri, Olov Rolandsson, Isabelle Romieu, Nina Roswall, Augustin Scalbert, Matthias Schulze, Nadia Slimani, Annemieke M. W. Spijkerman, Anne Tjonneland, Maria Jose Tormo, Marina Touillaud, Rosario Tumino, Daphne L. van der A, Geertruida J. van Woudenbergh, Claudia Langenberg, Elio Riboli, Nicholas J. Wareham

**Affiliations:** 5 MRC Epidemiology Unit, University of Cambridge, Institute of Metabolic Science, Cambridge, UK; 6 Unit of Nutrition, Environment, and Cancer, Catalan Institute of Oncology (ICO), Bellvitge Biomedical Research Institute (IDIBELL), Barcelona, Spain; 7 Department of Epidemiology, German Institute of Human Nutrition Potsdam–Rehbrücke, Nuthetal, Germany; 8 Public Health Institute of Navarra, Pamplona, Spain; 9 CIBER Epidemiology and Public Health (CIBERESP), Madrid, Spain; 10 Julius Center for Health Sciences and Primary Care, University Medical Center Utrecht, Utrecht, The Netherlands; 11 Public Health Department of Gipuzkoa, BioDonostia Research Institute, Health Department of the Basque Region, San Sebastián, Spain; 12 National Food Institute, Technical University of Denmark, Moerkhoej, Denmark; 13 INSERM, Centre for Research in Epidemiology and Population Health (CESP), U1018, Nutrition, Hormones and Women's Health, Villejuif, France; 14 Paris South University, UMRS 1018, Villejuif, France; 15 Division of Human Nutrition–Section of Nutrition and Epidemiology, University of Wageningen, Wageningen, The Netherlands; 16 Genetic and Molecular Epidemiology Unit, Clinical Research Center, Skåne University Hospital, Lund University, Malmö, Sweden; 17 Nutritional Epidemiology Unit, IRCCS Foundation National Institute of Oncology, Milan, Italy; 18 Department of Cancer Epidemiology, German Cancer Research Center, Heidelberg, Germany; 19 Cancer Epidemiology Unit, University of Oxford, Oxford, UK; 20 Department of Public Health and Primary Care, University of Cambridge, Cambridge, UK; 21 Molecular and Nutritional Epidemiology Unit, Cancer Research and Prevention Institute–ISPO, Florence, Italy; 22 Department of Clinical Medicine and Surgery, Federico II University, Naples, Italy; 23 Andalusian School of Public Health, Granada, Spain; 24 Department of Clinical Sciences, Lund University, Malmö, Sweden; 25 Section for Epidemiology, Department of Public Health, Aarhus University, Aarhus, Denmark; 26 Public Health and Health Planning Directorate, Asturias, Spain; 27 Human Genetic Foundation (HuGeF), Turin, Italy; 28 Department of Public Health and Clinical Medicine, Umeå University, Umeå, Sweden; 29 Section of Nutrition and Metabolism, International Agency for Research on Cancer (IARC), Lyon, France; 30 Institute of Cancer Epidemiology, Danish Cancer Society, Copenhagen, Denmark; 31 Department of Molecular Epidemiology, German Institute of Human Nutrition Potsdam–Rehbrücke, Nuthetal, Germany; 32 National Institute for Public Health and the Environment (RIVM), Bilthoven, The Netherlands; 33 Epidemiology Department, Murcia Regional Health Council, Murcia, Spain; 34 Department of Health and Social Sciences, Universidad de Murcia, Murcia, Spain; 35 Cancer Registry and Histopathology Unit, “Civile M.P. Arezzo” Hospital, Ragusa, Italy; and; 36 School of Public Health, Imperial College London, London, UK

## Abstract

Dietary flavanols and flavonols, flavonoid subclasses, have been recently associated with a lower risk of type 2 diabetes (T2D) in Europe. Even within the same subclass, flavonoids may differ considerably in bioavailability and bioactivity. We aimed to examine the association between individual flavanol and flavonol intakes and risk of developing T2D across European countries. The European Prospective Investigation into Cancer and Nutrition (EPIC)–InterAct case-cohort study was conducted in 8 European countries across 26 study centers with 340,234 participants contributing 3.99 million person-years of follow-up, among whom 12,403 incident T2D cases were ascertained and a center-stratified subcohort of 16,154 individuals was defined. We estimated flavonoid intake at baseline from validated dietary questionnaires using a database developed from Phenol-Explorer and USDA databases. We used country-specific Prentice-weighted Cox regression models and random-effects meta-analysis methods to estimate HRs. Among the flavanol subclass, we observed significant inverse trends between intakes of all individual flavan-3-ol monomers and risk of T2D in multivariable models (all *P*-trend < 0.05). We also observed significant trends for the intakes of proanthocyanidin dimers (HR for the highest vs. the lowest quintile: 0.81; 95% CI: 0.71, 0.92; *P-*trend = 0.003) and trimers (HR: 0.91; 95% CI: 0.80, 1.04; *P-*trend = 0.07) but not for proanthocyanidins with a greater polymerization degree. Among the flavonol subclass, myricetin (HR: 0.77; 95% CI: 0.64, 0.93; *P-*trend = 0.001) was associated with a lower incidence of T2D. This large and heterogeneous European study showed inverse associations between all individual flavan-3-ol monomers, proanthocyanidins with a low polymerization degree, and the flavonol myricetin and incident T2D. These results suggest that individual flavonoids have different roles in the etiology of T2D.

## Introduction

Prospective studies have shown that the consumption of plant-based foods, such as fruit and vegetables (1), tea (2), and wine (3, 4), is related to a lower risk of type 2 diabetes (T2D)^37^. Flavonoids and other polyphenolic compounds may play a relevant role in the health effects of plant-based diets, but the evidence is limited. Flavonoids are a large group of secondary metabolites in plants that comprise 6 subclasses: flavanols or flavan-3-ols (flavan-3-ol monomers, proanthocyanidins, and theaflavins), anthocyanidins, flavonols, flavanones, flavones, and isoflavones.

We recently reported that diets rich in flavonoids are associated with a lower incidence of T2D in the European Prospective Investigation into Cancer and Nutrition (EPIC)–InterAct study (5). In particular, flavanols (including flavan-3-ol monomers and theaflavins, but not proanthocyanidins) and flavonols were the flavonoid subclasses that were significantly inversely associated with T2D (5). Similar results for flavonol intake were reported in a Finnish study (6), whereas no association for these flavonoids was observed in studies conducted in the United States (3, 7, 8). As a result of considerable differences in their chemical structure, there are large differences in bioavailability (from 0.1% of some anthocyanidins to 50% of some isoflavones) and bioactivity between different types of flavonoids (9, 10). To our knowledge, only the Finnish (6) and 1 of the U.S. studies (8) have assessed the effect of the intake of some individual flavonoids (flavonols and flavanones, and flavonols and flavones, respectively) on the risk of T2D. An inverse trend between quercetin and myricetin intake and T2D incidence was observed only in the Finnish study (6). Therefore, distinction within different subclasses (individual flavonoids) is recommended in studies assessing their health effects.

The aim of the present study was to investigate the association between dietary individual flavanol and flavonol intakes, which are the 2 flavonoid subclasses previously related to T2D in the EPIC-InterAct study (5), and the risk of developing T2D in a Europe-wide population with a large heterogeneity in the intake of these compounds (11, 12).

## Participants and Methods

### 

#### Study design, population, and case ascertainment.

The rationale and design of the EPIC-InterAct study have been previously published (13). In short, EPIC-InterAct is a case-cohort study nested within the EPIC study (14), comprising cohorts recruited in the 1990s from 8 European countries (Denmark, France, Germany, Italy, The Netherlands, Spain, Sweden, and the United Kingdom) across 26 participating study centers (13). Detailed information on numbers by country and center was provided previously (13), but, in summary, of the 455,680 participants across the 8 countries of EPIC, after the exclusion of individuals without stored blood (*n* = 109,625) or without information on reported diabetes status (*n* = 5821), 340,234 participants with 3.99 million person-years of follow-up were eligible for this study. Ethical review boards of the International Agency for Research on Cancer and local participating centers approved the project.

We identified and verified cases of incident T2D by using multiple data sources of evidence, including self-report, linkage to primary care registers, secondary care registers, medication registers, hospital admissions, and mortality data (13). Follow-up was censored on 31 December 2007 or the date of death or T2D diagnosis, whichever occurred first. In total, we identified 12,403 verified incident cases of T2D.

We randomly selected a center-stratified subcohort of 16,835 individuals from the 340,234 eligible participants. After exclusion of participants with prevalent diabetes or unknown diabetes status (*n* = 681), 16,154 individuals were included in the subcohort. Because of random selection, this subcohort also included a random set of 778 individuals who developed incident T2D during follow-up. The case-cohort design allows for the random occurrence of incident cases in the subcohort and accounts for this in the statistical analysis.

Of the 27,779 participants, we excluded 619 participants with a ratio of energy intake to energy expenditure in the top and bottom 1% of the distribution and 1072 participants with missing information on dietary intake or other covariates used in the statistical analysis. The final analysis sample included 26,088 participants (11,559 cases and a subcohort of 15,258 participants, including 729 cases in the subcohort).

#### Data collection.

At baseline, usual dietary intake during the year before recruitment was measured by country-specific validated dietary questionnaires designed to reflect local dietary patterns (14, 15). We estimated total energy and nutrient intakes by using the standardized EPIC Nutrient Database (16).

We obtained baseline information on other lifestyle variables from face-to-face or self-administered and standardized questionnaires including questions on education, medical history, physical activity, and smoking (14, 17). We measured anthropometric data by using standardized protocols, except in Oxford (UK) and France, where we collected self-reported measurements (14).

#### Flavanol and flavonol intake.

Estimated flavanol and flavonol intakes were derived from foods included in the dietary questionnaires through a comprehensive food composition database on flavonoids as previously described (11, 12, 18). Our database on flavanols (flavan-3-ol monomers, proanthocyanidins, and theaflavins) and flavonols was based on USDA databases (19, 20) and Phenol-Explorer (21). This online database compiles composition data on individual flavan-3-ol monomers (catechin, epigallocatechin, epicatechin, epicatechin 3-gallate, epigallocatechin 3-gallate, gallocatechin, catechin 3-gallate), proanthocyanidin groups (dimers, trimers, 4–6 monomers, 7–10 monomers, polymers), and the individual flavonols (isorhamnetin, kaempferol, myricetin, quercetin). Data on flavonoids were expressed as aglycone equivalents, after conversion of the flavonoid glycosides into aglycone contents using their respective molecular weights. The final database contains 1877 food items, including raw foods, cooked foods, and recipes, and 10% of values for these food items are missing.

#### Statistical analysis.

We assessed the distributions of intakes of total and individual flavan-3-ol monomers, proanthocyanidins, theaflavins, and flavonols by using means, SDs, medians, and 5th and 95th percentiles because the data were skewed to the right. We used Spearman correlations to assess whether individual flavonoid intakes were correlated to each other within the same flavonoid subclass. The contribution of each food group to individual flavonoid intake was also computed as a percentage. We examined baseline characteristics and dietary intakes in the subcohort by quintiles of the sum of flavanol and flavonol intake. Prentice-weighted Cox regression models (22), which account for the case-cohort design, were used to estimate the associations between intakes of individual flavan-3-ol monomers, proanthocyanidins, and flavonols and risk of T2D. We categorized individual flavonoids by using subcohort-wide quintiles. Tests for linear trend were performed by assigning the medians of each quintile as scores. Intakes were also analyzed continuously after a log_2_ transformation so that a 1-unit increase represents a doubling of flavonoid intake. We estimated HRs and their 95% CIs by using the following modeling strategy based on already known confounding variables. Age was used as the underlying time scale, with entry time defined as the participant’s age at baseline and exit time as age at diagnosis of diabetes, censoring, or death (whichever came first). All models were stratified by center to control for center effects such as follow-up procedures and questionnaire design. Model 1 was adjusted for sex and total energy intake (kcal/d). Model 2 was additionally adjusted for educational level (none, primary school, technical/professional, secondary school, longer education), physical activity index (inactive, moderately inactive, moderately active, and active) (17), smoking status (never, former, and current), BMI (kg/m^2^), and alcohol intake (g/d). Model 3 was additionally adjusted for intakes of red meat, processed meat, sugar-sweetened soft drinks, and coffee (g/d). Model 4 was additionally adjusted for intakes of fiber (g/d), vitamin C (mg/d), and magnesium (mg/d). Models were not mutually adjusted for the rest of flavonoids within the subclass because of the high correlations between them (**Supplemental Tables 1–3**). We estimated HRs and their 95% CIs within each country and then combined them by using random-effects meta-analysis. Between-country heterogeneity was assessed by using the *I^2^* statistic (23). Effect modification by sex, baseline BMI category (BMI <25, 25 to < 30, and ≥30 kg/m^2^), and smoking status (never, former, current smokers) were assessed by modeling interaction terms between these variables and total intakes of flavan-3-ol monomers, proanthocyanidins, and flavonols.

We conducted sensitivity analyses excluding incident T2D cases diagnosed within the first 2 y of recruitment (*n* = 975). In a second sensitivity analysis, model 4 was additionally adjusted for self-reported hypertension and hyperlipidemia, after exclusion of 1971 participants with cancer and/or cardiovascular diseases at recruitment, because these subgroups may have modified their diet. In a third sensitivity analysis, model 4 was additionally adjusted for history of diabetes in a first-degree relative (after exclusion of 12,977 participants without these data), an important risk factor for T2D (24); and finally, model 4 was additionally adjusted for waist circumference (after exclusion of 1824 participants without these data), another independent risk factor strongly associated with T2D (25). All statistical analyses were performed by using Stata/SE 12.0 (StataCorp). All *P* values were based on 2-sided tests, and significance was set at *P* < 0.05.

## Results

**Table 1** shows the mean (SD), median, and percentile (5th and 95th) of individual flavan-3-ol monomer, proanthocyanidin, theaflavin, and flavonol intakes in the EPIC-InterAct subcohort. As indicated by the large differences between means and medians, particularly for flavan-3-ol monomers, the distributions were skewed to higher values. Epigallocatechin 3-gallate was the highest contributor (45.6%) to total flavan-3-ol monomer intake, followed by epicatechin 3-gallate (14.2%), epigallocatechin (13.8%), epicatechin (13.2%), catechin (9.2%), catechin 3-gallate (2.5%), and gallocatechin (1.5%). Polymers represented the subclass of proanthocyanidins that were most abundant (34.5%), followed by 4–6 monomers (22.2%), dimers (18.9%), 7–10 monomers (16.3%), and trimers (8.1%). Quercetin was the highest contributor to flavonols (70.2%), followed by kaempferol (18.5%), myricetin (9.3%), and isorhamnetin (2.0%). The main food sources of flavan-3-ol monomers, proanthocyanidins, and flavonols are shown in Table 1. Theaflavins were exclusively found in tea; therefore, the association with T2D was not considered. All flavan-3-ol monomer intakes were highly correlated with each other (Supplemental Table 1), as were proanthocyanidins (Supplemental Table 2) and flavonols, except for isorhamnetin (Supplemental Table 3).

**TABLE 1:** Dietary intake of flavanols and flavonols in the EPIC-InterAct subcohort^1^

Flavonoid	Mean ± SD	Median (5th, 95th percentile)	Main food sources
Flavanols, *mg/d*	334 ± 286	246 (60.9, 938)	Tea (39.1%), fruit (34.2%), wine (7.9%), chocolate (5.0%)
Flavan-3-ol monomers	146.2 ± 228.7	41.4 (9.2, 711.2)	Tea (81.0%), fruit (7.1%), wine (3.4%), chocolate (3.0%)
(−)-Epigallocatechin 3-gallate	66.7 ± 124.8	4.9 (0.2, 375.8)	Tea (97.0%), chocolate (1.6%), cakes (0.6%), fruit (0.5%)
(−)-Epicatechin 3-gallate	20.7 ± 36.7	3.0 (0.0, 110.3)	Tea (91.9%), herbal tea (5.5%), chocolate (1.2%), beer and cider (0.5%)
(−)-Epigallocatechin	20.2 ± 35.3	3.0 (0.5, 107.1)	Tea (91.3%), fruit (3.8%), beer and cider (2.5%), coffee (0.8%)
(−)-Epicatechin	19.3 ± 18.0	13.6 (3.2, 57.0)	Tea (40.4%), fruit (27.4%), chocolate (11.4%), wine (8.2%)
(+)-Catechin	13.5 ± 10.7	10.6 (2.7, 34.1)	Fruit (29.7%), wine (24.3%), tea (22.1%), beer and cider (9.8%)
(+)-Catechin 3-gallate	3.6 ± 6.8	0.3 (0.0, 20.3)	Tea (98.3%), herbal tea (1.7%)
(+)-Gallocatechin	2.2 ± 3.9	0.3 (0.0, 11.7)	Tea (93.8%), beer and cider (3.6%), fruit (0.4%), legumes (0.1%)
Proanthocyanidins	183 ± 140	151 (41.7, 423)	Fruit (56.8%), wine (11.7%), chocolate (6.8%), juices (4.5%)
Dimers	34.5 ± 29.5	26.8 (6.3, 86.4)	Fruit (38.0%), wine (25.7%), tea (17.8%), chocolate (5.6%)
Trimers	14.8 ± 12.3	12.1 (3.1, 34.7)	Fruit (52.2%), juices (11.4%), chocolate (8.8%), tea (7.4%)
4–6mers	40.6 ± 32.4	32.9 (8.5, 96.1)	Fruit (62.7%), chocolate (9.0%), wine (8.6%), juices (4.2%)
7–10mers	29.8 ± 24.8	23.8 (5.4, 73.5)	Fruit (64.9%), wine (8.7%), chocolate (6.1%), legumes (5.4%)
Polymers	63.0 ± 50.2	51.3 (12.5, 147.9)	Fruit (60.4%), wine (9.1%), legumes (7.8%), chocolate (5.9%)
Theaflavins	4.6 ± 8.8	0.08 (0, 26.4)	Tea (100%)
Flavonols, *mg/d*	24.8 ± 16.0	20.4 (7.8, 57.4)	Vegetables (27.2%), tea (26.4%), fruit (15.6%), wine (7.3%)
Quercetin	17.4 ± 10.0	15.1 (5.7, 36.8)	Vegetables (29.0%), fruit (21.1%), tea (20.3%), wine (6.6%)
Kaempferol	4.6 ± 5.1	2.6 (0.4, 15.1)	Tea (44.4%), vegetables (27.8%), beer and cider (17.4%), wine (2.8%)
Myricetin	2.3 ± 2.4	1.3 (0.3, 7.7)	Tea (51.8%), wine (22.1%), coffee (8.9%), vegetables (4.5%)
Isorhamnetin	0.5 ± 0.6	0.4 (0.1, 1.6)	Vegetables (58.8%), fruit (16.4%), wine (6.6%), herbal tea (4.2%)

1 *n* = 15,258. EPIC, European Prospective Investigation into Cancer and Nutrition; 4–6mers, 4–6 monomers; 7–10mers, 7–10 monomers.

Participants in the highest quintile of total flavanol and flavonol intakes were likely to be older with a greater educational level and with a more health-conscious lifestyle pattern (more physically active with lower BMI and tobacco consumption, higher intakes of fiber, vitamin C, magnesium, fruit, and vegetables; and a lower consumption of processed meat compared with those in the lowest quintile); however, participants in the top quintile reported greater alcohol and red meat intake, and a lower intake of coffee (**Table 2**). Participants across quintiles had similar frequencies of prevalent diseases.

**TABLE 2:** Baseline characteristics and dietary intakes of the EPIC-InterAct subcohort according to quintiles of sum of flavanol and flavonol intake^1^

		Quintile of sum of flavanols and flavonols				
Characteristic	All (*n* = 15,258)	1 (*n* = 3052)	2 (*n* = 3052)	3 (*n* = 3051)	4 (*n* = 3052)	5 (*n* = 3051)
Cutoff, *mg/d*		<139.8	139.8–217.5	217.6–321.7	321.8–526.0	>526.0
Median intake, *mg/d*		97.6	176.6	265.2	397.1	713.6
Sociodemographic characteristics						
Age, *y*	52.4 ± 9.1	52.1 ± 9.4	52.1 ± 9.0	51.7 ± 9.1	51.8 ± 8.6	54.2 ± 9.1
Men, *%*	37.8	40.3	35.8	34.6	38.7	39.8
Educational level, *%*						
None	7.7	7.6	8.7	9.6	8.4	4.1
Primary school	33.3	40.3	33.9	33.4	31.7	27
Technical/professional	23.2	24.5	22.6	21.6	21.7	25.8
Secondary school	15.1	12.3	13.8	15.7	16.6	17.2
Longer education	20.7	15.3	21.1	19.6	21.7	25.9
Anthropometric characteristics						
BMI, *kg/m^2^*	26.0 ± 4.2	26.2 ± 4.3	26.2 ± 4.3	26.0 ± 4.0	26.2 ± 4.2	25.5 ± 3.9
Waist circumference,^2^ *cm*	86.4 ± 12.6	87.2 ± 12.9	86.5 ± 12.8	85.9 ± 12.5	86.9 ± 12.4	86.4 ± 12.6
Lifestyle characteristics						
Smoking status, *%*						
Never	46.8	39.3	45.3	51.2	50.1	48.3
Former	27.2	23.4	26.0	25.0	28.9	32.5
Current	26.0	37.3	28.7	23.8	20.9	19.2
Physical activity, *%*						
Inactive	23.6	27.5	26.3	24.0	21.4	18.9
Moderately inactive	33.7	33.9	34.0	33.5	35.3	31.5
Moderately active	22.7	21.5	20.5	23.8	22.4	25.1
Active	20.1	17.0	19.3	18.7	20.9	24.4
Prevalent diseases, yes, *%*						
Cancer	3.2	3.6	3.7	2.8	3.1	3.1
Myocardial infarction^2^	1.4	1.8	1.8	0.9	1.0	1.5
Stroke^2^	0.9	1.3	1.0	0.6	0.6	0.8
Angina^2^	2.1	2.0	2.4	1.8	1.6	2.4
Hypertension^2^	18.6	18.3	20.0	19.4	18.0	17.2
Hyperlipidemia^2^	17.3	15.5	18.1	19.9	19.2	13.7
Family history of diabetes^2^	19.2	19.8	18.9	20.4	21.7	16.5
Dietary intake						
Total energy, *kcal/d*	2140 ± 635	1920 ± 575	2080 ± 594	2170 ± 624	2260 ± 650	2260 ± 661
Alcohol, *g/d*	13.2 ± 18.5	8.8 ± 13.0	12.0 ± 16.8	13.1 ± 17.9	15.7 ± 20.1	16.4 ± 22.4
Fiber, *g/d*	22.8 ± 7.8	18.1 ± 5.9	21.3 ± 6.4	23.0 ± 6.8	25.3 ± 7.5	26.3 ± 8.9
Vitamin C, *mg/d*	124 ± 68	88 ± 52	116 ± 58	128 ± 61	145 ± 68	142 ± 79
Magnesium, *mg/d*	351 ± 103	310 ± 91	340 ± 97	352 ± 103	368 ± 104	384 ± 105
Red meat, *g/d*	46 ± 36	45 ± 36	45 ± 35	44 ± 35	46 ± 34	50 ± 40
Processed meat, *g/d*	37 ± 32	38 ± 31	40 ± 34	38 ± 33	36 ± 33	32 ± 31
Soft drinks, *g/d*	69 ± 155	76 ± 175	71 ± 154	65 ± 157	57 ± 127	74 ± 158
Coffee, *g/d*	384 ± 385	496 ± 436	433 ± 405	350 ± 365	303 ± 327	337 ± 349
Fruit, *g/d*	234 ± 188	109 ± 91	190 ± 116	250 ± 151	319 ± 196	305 ± 253
Vegetables, *g/d*	183 ± 119	139 ± 103	173 ± 109	183 ± 115	200 ± 122	219 ± 128

1 Values are means ± SDs or percentages. EPIC, European Prospective Investigation into Cancer and Nutrition.

2 Missing data: waist circumference (*n* = 1013), myocardial infarction (*n* = 230), stroke (*n* = 1209), angina (*n* = 5139), hypertension (*n* = 45), hyperlipidemia (*n* = 2944), family history of diabetes (*n* = 7643). Prevalent diseases were self-reported.

The pooled HRs (95% CIs) for T2D comparing quintiles of individual flavan-3-ol monomer, proanthocyanidin, and flavonol intakes are shown in **Tables 3–5**, respectively. We observed statistically significant inverse associations in model 1 [stratified by center and adjusted for age (as underlying time scale), sex, and total energy] for all individual compounds, except for the flavonol isorhamnetin. After further adjustment for potential confounders (models 2 and 3), all associations were attenuated. When we additionally included fiber, vitamin C, and magnesium in the multivariable models (model 4), we observed significant inverse trends between incidence of T2D and all individual flavan-3-ol monomers, although the HR for the highest versus the lowest quintile was only significant for epigallocatechin 3-gallate (HR: 0.64; 95% CI: 0.44, 0.92), catechin (HR: 0.86; 95% CI: 0.75, 0.99), catechin 3-gallate (HR comparing extreme quantiles: 0.80; 95% CI: 0.69, 0.93), and gallocatechin (HR: 0.71; 95% CI: 0.59, 0.85). For proanthocyanidins, we found a significant inverse association with dimers (HR: 0.81; 95% CI: 0.71, 0.92; *P*-trend = 0.003) and a borderline inverse trend with trimers (HR: 0.91; 95% CI: 0.80, 1.04; *P*-trend = 0.07). For flavonols, we found a significant inverse association with myricetin (HR: 0.77; 95% CI: 0.64, 0.93; *P*-trend = 0.001) and a significant trend with kaempferol (HR: 0.91; 95% CI: 0.78, 1.05; *P*-trend = 0.013).

**TABLE 3:** Pooled HRs (95% CIs) for the association between flavan-3-ol monomer intakes and type 2 diabetes: the EPIC-InterAct study^1^

Flavan-3-ol monomer	Quintile 1	Quintile 2	Quintile 3	Quintile 4	Quintile 5	*P*-trend^2^	Continuous (log_2_)^3^
(−)-Epigallocatechin 3-gallate, *mg/d*	<0.87	0.87–2.40	2.41–11.64	11.65–108.77	>108.77		
Median intake, *mg/d*	0.4	1.46	4.9	40.8	219.62		
Model 1	1 (ref)	0.83 (0.73, 0.95)	0.78 (0.66, 0.94)	0.73 (0.59, 0.90)	0.57 (0.44, 0.73)	<0.001	0.96 (0.94, 0.98)
Model 2	1 (ref)	0.85 (0.74, 0.98)	0.89 (0.70, 1.13)	0.82 (0.63, 1.07)	0.69 (0.47, 1.01)	0.24	0.99 (0.97, 1.01)
Model 3	1 (ref)	0.86 (0.74, 1.00)	0.88 (0.69, 1.12)	0.80 (0.61, 1.04)	0.64 (0.45, 0.92)	0.008	0.98 (0.96, 1.00)
Model 4	1 (ref)	0.85 (0.74, 0.99)	0.87 (0.69, 1.11)	0.79 (0.60, 1.03)	0.64 (0.44, 0.92)	0.012	0.98 (0.96, 1.00)
(−)-Epicatechin 3-gallate, *mg/d*	<0.31	0.31–1.34	1.35–6.17	6.18–32.21	>32.21		
Median intake, *mg/d*	0.11	0.6	2.98	15.08	64.48		
Model 1	1 (ref)	0.88 (0.79, 0.98)	0.86 (0.73, 1.02)	0.77 (0.63, 0.94)	0.64 (0.51, 0.81)	<0.001	0.96 (0.95, 0.98)
Model 2	1 (ref)	1.00 (0.89, 1.11)	1.01 (0.81, 1.25)	0.96 (0.79, 1.17)	0.88 (0.66, 1.18)	0.54	1.00 (0.98, 1.01)
Model 3	1 (ref)	0.99 (0.88, 1.13)	0.99 (0.79, 1.23)	0.92 (0.74, 1.14)	0.80 (0.60, 1.06)	0.024	0.99 (0.97, 1.00)
Model 4	1 (ref)	1.00 (0.87, 1.15)	0.98 (0.77, 1.24)	0.90 (0.71, 1.15)	0.80 (0.59, 1.08)	0.031	0.98 (0.97, 1.00)
(−)-Epigallocatechin, *mg/d*	<1.09	1.09–2.05	2.06–5.33	5.34–31.36	>31.36		
Median intake, *mg/d*	0.73	1.5	3.04	13.66	63.06		
Model 1	1 (ref)	0.85 (0.78, 0.92)	0.87 (0.75, 1.01)	0.83 (0.69, 0.99)	0.64 (0.53, 0.79)	<0.001	0.95 (0.94, 0.97)
Model 2	1 (ref)	0.92 (0.81, 1.04)	0.98 (0.84, 1.14)	1.01 (0.85, 1.19)	0.81 (0.63, 1.05)	0.52	0.99 (0.97, 1.01)
Model 3	1 (ref)	0.95 (0.83, 1.08)	1.01 (0.86, 1.18)	1.02 (0.86, 1.21)	0.80 (0.63, 1.01)	0.020	0.98 (0.96, 1.00)
Model 4	1 (ref)	0.95 (0.85, 1.07)	1.00 (0.84, 1.19)	1.01 (0.83, 1.21)	0.79 (0.61, 1.03)	0.022	0.98 (0.96, 1.00)
(−)-Epicatechin, *mg/d*	<6.76	6.76–11.02	11.03–16.83	16.84–28.75	>28.75		
Median intake, *mg/d*	4.56	8.81	13.62	21.02	41.35		
Model 1	1 (ref)	0.90 (0.80, 1.01)	0.77 (0.68, 0.88)	0.73 (0.63, 0.85)	0.67 (0.53, 0.85)	<0.001	0.87 (0.82, 0.92)
Model 2	1 (ref)	0.98 (0.87, 1.10)	0.91 (0.82, 1.01)	0.89 (0.80, 1.00)	0.92 (0.77, 1.09)	0.24	0.95 (0.91, 0.99)
Model 3	1 (ref)	0.99 (0.88, 1.11)	0.92 (0.83, 1.02)	0.89 (0.77, 1.02)	0.87 (0.71, 1.06)	0.05	0.93 (0.89, 0.98)
Model 4	1 (ref)	0.99 (0.89, 1.11)	0.92 (0.82, 1.03)	0.87 (0.75, 1.01)	0.84 (0.69, 1.04)	0.040	0.93 (0.89, 0.98)
(+)-Catechin, *mg/d*	<5.50	5.50–8.79	8.80–12.78	12.79–20.08	>20.08		
Median intake, *mg/d*	3.81	7.11	10.59	15.65	27.02		
Model 1	1 (ref)	0.84 (0.73, 0.97)	0.75 (0.66, 0.86)	0.67 (0.57, 0.80)	0.64 (0.51, 0.80)	<0.001	0.84 (0.78, 0.91)
Model 2	1 (ref)	1.01 (0.87, 1.17)	0.92 (0.82, 1.03)	0.94 (0.84, 1.06)	0.98 (0.78, 1.02)	0.024	0.96 (0.93 0.99)
Model 3	1 (ref)	1.02 (0.88, 1.18)	0.93 (0.81, 1.06)	0.94 (0.84, 1.06)	0.87 (0.76, 1.00)	0.006	0.94 (0.90, 0.98)
Model 4	1 (ref)	1.01 (0.87, 1.18)	0.92 (0.79, 1.07)	0.93 (0.83, 1.05)	0.86 (0.75, 0.99)	0.005	0.94 (0.91, 0.98)
(+)-Catechin 3-gallate^4^, *mg/d*	0	0.01–0.50	0.51–5.65	>5.65	—		
Median intake, *mg/d*	0	0.19	2.03	11.85	—		
Model 1	1 (ref)	0.88 (0.77, 0.99)	0.84 (0.74, 0.95)	0.65 (0.57, 0.76)	—	<0.001	0.99 (0.98, 0.99)
Model 2	1 (ref)	0.99 (0.85, 1.16)	0.96 (0.86, 1.07)	0.85 (0.72, 1.02)	—	0.29	1.00 (0.99, 1.00)
Model 3	1 (ref)	0.99 (0.85, 1.15)	0.93 (0.83, 1.04)	0.78 (0.67, 0.91)	—	0.006	1.00 (0.99, 1.00)
Model 4	1 (ref)	0.98 (0.84, 1.15)	0.93 (0.82, 1.05)	0.80 (0.69, 0.93)	—	0.009	1.00 (0.99, 1.00)
(+)-Gallocatechin, *mg/d*	<0.04	0.04–0.18	0.19–0.59	0.60–3.45	>3.45		
Median intake, *mg/d*	0.015	0.09	0.31	1.48	7.01		
Model 1	1 (ref)	0.79 (0.69, 0.91)	0.78 (0.72, 0.86)	0.74 (0.62, 0.89)	0.58 (0.50, 0.68)	<0.001	0.96 (0.95, 0.97)
Model 2	1 (ref)	0.84 (0.65, 1.09)	0.88 (0.78, 0.99)	0.86 (0.70, 1.05)	0.75 (0.63, 0.89)	0.57	0.99 (0.97, 1.00)
Model 3	1 (ref)	0.87 (0.68, 1.12)	0.89 (0.80, 1.01)	0.85 (0.68, 1.06)	0.71 (0.59, 0.85)	0.022	0.98 (0.97, 0.99)
Model 4	1 (ref)	0.88 (0.68, 1.13)	0.89 (0.79, 1.00)	0.84 (0.68, 1.05)	0.71 (0.59, 0.85)	0.027	0.98 (0.97, 0.99)

1 For model 1, the pooled HRs were based on random-effects meta-analysis by using Prentice-weighted Cox regression analysis, stratified by center and adjusted for sex and total energy intake. Model 2 was additionally adjusted for educational level, smoking status, physical activity levels, BMI, and alcohol intake. Model 3 was additionally adjusted for red meat, processed meat, sugar-sweetened soft drinks, and coffee intakes. Model 4 was additionally adjusted for fiber, vitamin C, and magnesium intakes. EPIC, European Prospective Investigation into Cancer and Nutrition; ref, reference.

2 Obtained by assigning the median of each quintile as scores.

3 A 1-unit increase represents a doubling of flavan-3-ol monomer intake.

4 Catechin 3-gallates were assessed in 4 groups because there was a large group of nonconsumers, which resulted in an unbalanced division of catechin 3-gallates in quintiles: group 1, *n* = 9499 (36.4%); group 2, *n* = 5930 (22.7%); group 3, *n* = 5621 (21.6%); group 4, *n* = 5038 (19.3%).

**TABLE 4:** Pooled HRs (95% CIs) for the association between proanthocyanidin intakes and type 2 diabetes: the EPIC-InterAct study^1^

Proanthocyanidin	Quintile 1	Quintile 2	Quintile 3	Quintile 4	Quintile 5	*P*-trend^2^	Continuous (log_2_)^3^
Dimers, *mg/d*	<14.1	14.1–22.1	22.2–32.3	33.4–49.5	>49.5		
Median intake, *mg/d*	9.33	17.92	26.82	39.65	66.52		
Model 1	1 (ref)	0.81 (0.73, 0.91)	0.75 (0.66, 0.85)	0.70 (0.59, 0.83)	0.66 (0.54, 0.81)	<0.001	0.86 (0.80, 0.92)
Model 2	1 (ref)	0.85 (0.73, 1.00)	0.89 (0.77, 1.03)	0.92 (0.82, 1.03)	0.85 (0.75, 0.96)	0.010	0.96 (0.93, 0.99)
Model 3	1 (ref)	0.85 (0.73, 0.99)	0.88 (0.75, 1.04)	0.89 (0.76, 1.03)	0.83 (0.73, 0.94)	0.003	0.94 (0.90, 0.99)
Model 4	1 (ref)	0.84 (0.72, 0.99)	0.87 (0.74, 1.02)	0.87 (0.74, 1.02)	0.81 (0.71, 0.92)	0.003	0.94 (0.90, 0.99)
Trimers, *mg/d*	<6.6	6.6–10.2	10.3–14.2	14.3–20.6	>20.6		
Median intake, *mg/d*	4.4	8.36	12.12	16.79	27.03		
Model 1	1 (ref)	0.87 (0.80, 0.94)	0.82 (0.73, 0.92)	0.78 (0.69, 0.88)	0.79 (0.69, 0.91)	<0.001	0.92 (0.88, 0.97)
Model 2	1 (ref)	0.94 (0.84, 1.06)	0.92 (0.79, 1.07)	0.96 (0.86, 1.07)	0.92 (0.82, 1.04)	0.045	0.97 (0.94, 1.00)
Model 3	1 (ref)	0.93 (0.84, 1.04)	0.90 (0.76, 1.07)	0.93 (0.80, 1.08)	0.92 (0.80, 1.06)	0.09	0.97 (0.93, 1.01)
Model 4	1 (ref)	0.93 (0.84, 1.03)	0.90 (0.77, 1.05)	0.93 (0.81, 1.07)	0.91 (0.80, 1.04)	0.07	0.97 (0.94, 1.01)
4–6mers, *mg/d*	<17.8	17.8–27.6	27.7–39.0	39.1–58.0	>58.0		
Median intake, *mg/d*	11.92	22.67	32.9	46.57	78.33		
Model 1	1 (ref)	0.87 (0.80, 0.94)	0.81 (0.74, 0.88)	0.81 (0.74, 0.89)	0.81 (0.73, 0.90)	0.001	0.92 (0.88, 0.97)
Model 2	1 (ref)	0.90 (0.82, 1.00)	0.89 (0.80, 1.00)	0.93 (0.83, 1.04)	0.88 (0.78, 1.00)	0.026	0.96 (0.93, 1.00)
Model 3	1 (ref)	0.91 (0.82, 1.01)	0.90 (0.80, 1.01)	0.95 (0.85, 1.07)	0.91 (0.80, 1.03)	0.12	0.97 (0.93, 1.00)
Model 4	1 (ref)	0.91 (0.82, 1.01)	0.89 (0.80, 1.00)	0.96 (0.85, 1.08)	0.92 (0.80, 1.05)	0.15	0.97 (0.95, 1.00)
7–10mers, *mg/d*	<2.3	12.3–19.6	19.6–28.7	28.8–42.8	>42.8		
Median intake, *mg/d*	7.81	15.92	23.76	34.31	59.18		
Model 1	1 (ref)	0.86 (0.78, 0.95)	0.83 (0.75, 0.92)	0.80 (0.72, 0.89)	0.83 (0.73, 0.93)	0.001	0.94 (0.91, 0.98)
Model 2	1 (ref)	0.92 (0.79, 1.07)	0.93 (0.82, 1.06)	0.91 (0.78, 1.05)	0.89 (0.78, 1.03)	0.030	0.98 (0.95, 1.00)
Model 3	1 (ref)	0.93 (0.80, 1.07)	0.94 (0.82, 1.08)	0.93 (0.80, 1.07)	0.92 (0.80, 1.07)	0.13	0.98 (0.96, 1.01)
Model 4	1 (ref)	0.92 (0.80, 1.06)	0.94 (0.82, 1.07)	0.94 (0.84, 1.06)	0.93 (0.81, 1.07)	0.15	0.98 (0.96, 1.01)
Polymers, *mg/d*	>27.9	27.9–43.2	43.3–61.0	61.1–90.5	>90.5		
Median intake, *mg/d*	18.78	35.57	51.35	72.95	118.27		
Model 1	1 (ref)	0.86 (0.78, 0.95)	0.79 (0.72, 0.87)	0.81 (0.74, 0.89)	0.81 (0.71, 0.92)	0.003	0.94 (0.90, 0.98)
Model 2	1 (ref)	0.85 (0.75, 0.97)	0.86 (0.74, 1.01)	0.90 (0.81, 1.01)	0.88 (0.76, 1.00)	0.09	0.97 (0.94, 1.00)
Model 3	1 (ref)	0.85 (0.77, 0.94)	0.86 (0.74, 1.01)	0.92 (0.82, 1.03)	0.90 (0.80, 1.03)	0.31	0.98 (0.95, 1.01)
Model 4	1 (ref)	0.85 (0.77, 0.94)	0.85 (0.74, 0.98)	0.93 (0.82, 1.05)	0.92 (0.80, 1.06)	0.42	0.98 (0.96, 1.01)

1 For model 1, the pooled HRs were based on random-effects meta-analysis by using Prentice-weighted Cox regression analysis, stratified by center and adjusted for sex and total energy intake. Model 2 was additionally adjusted for educational level, smoking status, physical activity levels, BMI, and alcohol intake. Model 3 was additionally adjusted for red meat, processed meat, sugar-sweetened soft drink, and coffee intakes. Model 4 was additionally adjusted for fiber, vitamin C, and magnesium intakes. EPIC, European Prospective Investigation into Cancer and Nutrition; ref, reference; 4–6mers, 4–6 monomers; 7–10mers, 7–10 monomers.

2 Obtained by assigning the median of each quintile as scores.

3 A 1-unit increase represents a doubling of proanthocyanidin intake.

**TABLE 5:** Pooled HRs (95% CIs) for the association between flavonol intakes and type 2 diabetes: the EPIC-InterAct study^1^

Flavonol	Quintile 1	Quintile 2	Quintile 3	Quintile 4	Quintile 5	*P*-trend^2^	Continuous (log_2_)^3^
Quercetin, *mg/d*	<9.28	9.28–13.00	13.01–17.48	17.49–24.37	>24.37		
Median intake, *mg/d*	7.14	11.11	15.06	20.53	31.12		
Model 1	1 (ref)	0.92 (0.85, 1.00)	0.82 (0.74, 0.90)	0.77 (0.65, 0.90)	0.75 (0.58, 0.97)	0.012	0.85 (0.77, 0.94)
Model 2	1 (ref)	1.02 (0.92, 1.12)	0.92 (0.80, 1.05)	0.90 (0.79, 1.01)	0.99 (0.87, 1.14)	0.24	0.95 (0.90, 1.01)
Model 3	1 (ref)	1.03 (0.93, 1.14)	0.92 (0.79, 1.06)	0.89 (0.76, 1.04)	0.93 (0.77, 1.13)	0.14	0.93 (0.85, 1.01)
Model 4	1 (ref)	1.02 (0.92, 1.13)	0.91 (0.79, 1.04)	0.86 (0.71, 1.05)	0.91 (0.74, 1.11)	0.15	0.92 (0.85, 1.00)
Kaempferol, *mg/d*	<1.00	1.00–1.92	1.93–3.59	3.60–7.57	>7.57		
Median intake, *mg/d*	0.65	1.41	2.62	5.14	12.18		
Model 1	1 (ref)	0.94 (0.87, 1.02)	0.93 (0.78, 1.10)	0.83 (0.74, 0.94)	0.75 (0.63, 0.89)	<0.001	0.93 (0.90, 0.97)
Model 2	1 (ref)	0.99 (0.90, 1.10)	1.08 (0.93, 1.25)	0.95 (0.84, 1.07)	0.96 (0.84, 1.11)	0.20	0.99 (0.96, 1.01)
Model 3	1 (ref)	1.00 (0.91, 1.11)	1.08 (0.94, 1.25)	0.93 (0.83, 1.05)	0.91 (0.79, 1.05)	0.010	0.97 (0.94, 1.00)
Model 4	1 (ref)	0.99 (0.90, 1.10)	1.08 (0.93, 1.25)	0.92 (0.82, 1.04)	0.91 (0.78, 1.05)	0.013	0.97 (0.94, 1.00)
Myricetin, *mg/d*	<0.57	0.57–1.01	1.02–1.74	1.75–3.69	>3.69		
Median intake, *mg/d*	0.37	0.78	1.32	2.43	5.38		
Model 1	1 (ref)	0.82 (0.73, 0.92)	0.72 (0.64, 0.81)	0.62 (0.53, 0.73)	0.55 (0.41, 0.75)	<0.001	0.86 (0.82, 0.91)
Model 2	1 (ref)	0.91 (0.74, 1.10)	0.88 (0.79, 0.99)	0.76 (0.67, 0.87)	0.75 (0.63, 0.91)	0.012	0.93 (0.90, 0.96)
Model 3	1 (ref)	0.96 (0.79, 1.18)	0.93 (0.82, 1.06)	0.80 (0.70, 0.91)	0.78 (0.65, 0.94)	0.001	0.92 (0.89, 0.96)
Model 4	1 (ref)	0.97 (0.79, 1.19)	0.94 (0.81, 1.08)	0.80 (0.70, 0.91)	0.77 (0.64, 0.93)	0.001	0.92 (0.88, 0.96)
Isorhamnetin, *mg/d*	<0.17	0.17–0.29	0.30–0.44	0.45–0.81	>0.81		
Median intake, *mg/d*	0.12	0.23	0.36	0.58	1.24		
Model 1	1 (ref)	0.86 (0.76, 0.97)	0.83 (0.70, 0.97)	0.76 (0.61, 0.95)	0.84 (0.62, 1.13)	0.17	0.92 (0.85, 1.00)
Model 2	1 (ref)	0.89 (0.77, 1.02)	0.84 (0.69, 1.03)	0.81 (0.63, 1.03)	0.92 (0.73, 1.17)	0.46	0.95 (0.89, 1.02)
Model 3	1 (ref)	0.87 (0.76, 1.00)	0.84 (0.69, 1.03)	0.81 (0.64, 1.03)	0.95 (0.73, 1.22)	0.69	0.96 (0.90, 1.02)
Model 4	1 (ref)	0.96 (0.73, 1.00)	0.83 (0.67, 1.05)	0.82 (0.64, 1.04)	0.97 (0.75, 1.25)	0.96	0.96 (0.90, 1.02)

1 For model 1, the pooled HRs were based on random-effects meta-analysis by using Prentice-weighted Cox regression analysis, stratified by center and adjusted for sex and total energy intake. Model 2 was additionally adjusted for educational level, smoking status, physical activity levels, BMI, and alcohol intake. Model 3 was additionally adjusted for red meat, processed meat, sugar-sweetened soft drink, and coffee intakes. Model 4 was additionally adjusted for fiber, vitamin C, and magnesium intakes. EPIC, European Prospective Investigation into Cancer and Nutrition; ref, reference.

2 Obtained by assigning the median of each quintile as scores.

3 A 1-unit increase represents a doubling of flavonol intake.

In multivariable analyses (model 4), we observed similar associations of T2D when dietary flavan-3-ol monomer, proanthocyanidin, and flavonol exposures were assessed as a continuous variable after log_2_ transformation (Tables 3–5). We detected no significant heterogeneity between countries for the associations of flavan-3-ol monomers (*I^2^* = 19.4%, *P* = 0.28), proanthocyanidins (*I^2^* = 0.0%, *P* = 0.77), and flavonols (*I^2^* = 26.8%, *P* = 0.21) with T2D. For intakes of flavan-3-ol monomers, proanthocyanidins, and flavonols, we found no interactions with sex (*P*-interaction = 0.44, 0.45, and 0.12, respectively), BMI (*P*-interaction = 0.75, 0.73, and 0.83, respectively), or smoking status (*P*-interaction = 0.18, 0.79, and 0.23, respectively).

In sensitivity analyses, we observed similar results after the exclusion of T2D cases diagnosed within the first 2 y of follow-up or participants with prevalent cardiovascular diseases. When family history of diabetes was added in model 4, associations were strengthened. After further adjustment for waist circumference (model 4), the findings were almost identical (data not shown).

## Discussion

In this large prospective study across 8 European countries, all flavan-3-ol monomers, proanthocyanidins with lower degree of polymerization, and the flavonol myricetin were inversely related to a lower risk of T2D. We found inverse trends between all individual flavan-3-ol monomer intakes and risk of T2D. Furthermore, among them, intakes of catechin, epigallocatechin 3-gallate, gallocatechin, and catechin 3-gallate were significantly inversely associated with incident T2D comparing extreme quantiles. Indeed, the main food sources of flavan-3-ol monomers (tea and some fruit, particularly apples and pears) in the EPIC study (11) were also inversely associated with incidence of T2D in several prospective studies (1, 2, 7, 26). In contrast to our findings, no associations between flavanols and T2D were reported in any of the previous cohorts conducted in the United States (3, 7). Differences in the range of intakes between countries could partially explain these inconsistencies. In the present European study, the median of flavan-3-ol monomer intake was 41.4 mg/d (10th–90th percentiles: 12.9–428.9 mg/d), whereas in 1 of the U.S. studies the median was 27.0 mg/d (10th–90th percentiles: 8.4–135.1 mg/d) (7). Several in vitro and in vivo studies have evaluated the antidiabetic effects of individual flavan-3-ol monomers and flavan-3-ol–rich foods (e.g., cocoa and tea), showing a high range of activities related to improving glucose homeostasis, such as inhibition of glucosidase activity and glucose absorption from the intestine, protection of pancreatic β cells, increased insulin secretion, activation of insulin receptors and glucose uptake in the insulin-sensitive tissues, and modulation of intracellular signaling pathways and genes involved in gluconeogenesis and glycogenesis (27–29).

As reported earlier, in the EPIC-InterAct study (5) and in a U.S.-based study (3), total proanthocyanidin subclass intake was not significantly related to T2D. However, examining the relations of dietary proanthocyanidins according to polymerization degree in the current study, a significant inverse association was observed between proanthocyanidin dimer intakes and risk of T2D. Moreover, a potential trend with proanthocyanidin trimer intake was found, but not with PAs with higher polymerization degrees. These results might be interpreted in 3 different ways. First, proanthocyanidin dimers are more bioactive than proanthocyanidin polymers. In the gut, proanthocyanidins inhibit the glucosidase activity and the formation of advanced glycation endproducts in an inverse polymerization degree manner; however, proanthocyanidin polymers showed a stronger inhibitory activity against α-amylase than did proanthocyanidin oligomers (30). Second, proanthocyanidin dimers and trimers are more bioavailable than polymers, and, as such, they can be better absorbed in the gut than proanthocyanidin polymers (9). Several pharmacokinetic studies have shown that proanthocyanidin polymers may not be degraded to monomers in the stomach (31) and polymerization greatly impairs intestinal absorption (9). In experimental studies, proanthocyanidin dimers improved insulin concentrations in the blood and pancreas (32). In addition, proanthocyanidins were able to regulate microRNA expression in human HepG2 cells, suggesting a new antidiabetic mechanism of action of proanthocyanidins (33). Third, proanthocyanidins not absorbed in the gut are partially hydrolyzed in the colon by the intestinal microbiota, taking into account that their degradation into phenolic acids decreases as the degree of polymerization increases (34). After proanthocyanidin hydrolyzation, phenolic acids can be absorbed in the colon, and then those may exert their antidiabetic effects, such as increasing insulin secretion, improving glucose uptake in muscle cells, and inducing hepatic glucokinase activity (28). These findings suggest a different effect of proanthocyanidins by polymerization degree in T2D, highlighting that a role in the prevention of T2D for proanthocyanidins may be confined to dimers and probably trimers but not to proanthocyanidins with a greater polymerization degree.

Among flavonols, myricetin was significantly inversely associated with T2D, and kaempferol tended to be inversely related to T2D risk. Similar results were reported for kaempferol and quercetin in the Finnish study (6), but no significant associations were observed for flavonol intakes in the U.S. studies (3, 7, 8). Surprisingly, in our study, quercetin, which was the most abundant contributor (70%) to flavonol intake, was not associated with T2D, although we note that quercetin intake was highly correlated with other flavonol intakes, except for intakes of isorhamnetin. These nonsignificant results could be explained by the wide CIs and the moderate heterogeneity observed among countries in our pooled analysis (*I^2^* = 44.5%, *P* = 0.08). This heterogeneity may be related to the large variability in quercetin intake among participating countries (12). According to flavonol dietary sources (12), tea [a food significantly associated with a lower incidence of T2D (2, 26)] was the main contributor to myricetin and kaempferol, whereas vegetables and fruit were the main food sources of quercetin, which are modestly associated with a lower incidence of T2D (1). To our knowledge, all individual flavonols are able to inhibit the activity of digestive enzymes for glucose production, particularly α-amylase, as well as of the transporters responsible for glucose absorption [sodium-glucose cotransporter 1 (SGLT1) and glucose transporter 2 (GLUT2)] (28, 35), although some differences in dose-response effects between individual flavonols may occur. Furthermore, enhanced pancreatic β-cell function and antioxidant, anti-inflammatory, and antiangiogenic activities of flavonols, through the regulation of signal transduction and different enzyme systems, may also be involved in their potential role against T2D (36, 37).

Limitations in our study included the use of a baseline assessment of diet and other lifestyle variables. Therefore, changes in lifestyle could not be taken into account in these analyses. In addition, measurement error in collecting self-reported dietary intake is inevitable. To minimize this, we used country-specific validated questionnaires for main food groups and nutrients (14, 15), although these have not been specifically validated for the intake of flavonoids. Moreover, flavanol and flavonol intake may be underestimated, although our database was mostly complete for these flavonoid subclasses (11, 12), and herb/plant supplement intakes were omitted in these analyses (up to 5% in Denmark, the highest consuming country) (38). Nutritional biomarkers offer an alternative method for estimating dietary intake that is objective rather than subjective, and they provide more accurate measures than self-reported questionnaires. To date, there are few validated biomarkers of flavanol and flavonol intakes, so further research in this field is warranted (39, 40). The association of dietary flavanol and flavonol intakes with T2D risk is likely susceptible to confounding because high flavanol and flavonol intake reflects a healthier lifestyle. In our models, we have adjusted for other determinants of a healthy lifestyle; however, possible residual confounding cannot be excluded. Finally, we realize that our study is prone to the well-known drawback of multiple comparisons, although Bender and Lange (41) concluded that adjustments for multiple testing are not necessary in exploratory studies such as this.

Strengths of the current study include the multicenter design and the large sample size at recruitment, from whom a large number of verified incident cases of T2D accrued during 3.99 million person-years of follow-up. This study also included a wide variation in flavanol and flavonol intakes among participants in 8 European countries. Furthermore, we were able to explore potential effect modifications and control for a number of plausible confounders and factors that may hide the etiologic pathway of the association between the intake of individual flavanols and flavonols and T2D. In all sensitivity analysis, the associations were almost identical, denoting the robustness of our results.

In conclusion, this large, prospective case-cohort study supports a protective role for all individual flavan-3-ol monomers, proanthocyanidins of low polymerization degree, and the flavonol myricetin against T2D in men and women across European countries. These results highlight the importance of the assessment of individual flavonoids in addition to that of the flavonoid subclasses. More studies in different populations are needed to confirm these potential inverse associations between the intake of individual flavanols and flavonols and the risk of developing T2D.

## Supplementary Material

Online Supporting Material

## Footnotes

37 Abbreviations used: EPIC, European Prospective Investigation into Cancer and Nutrition; GLUT2, glucose transporter 2; SGLT1, sodium-glucose cotransporter 1; T2D, type 2 diabetes.
