# Supplementary material for: Dietary Intakes of Individual Flavanols and Flavonols Are Inversely Associated with Incident Type 2 Diabetes in European Populations
Source: J Nutr. 2013 Dec 24;144(3):335–43. doi: 10.3945/jn.113.184945 (PMC3927546; doi:10.3945/jn.113.184945)
Supplement: Online Supporting Material [file supp_144_3_335__index.html]

Online Supporting Material 

# Dietary Intakes of Individual Flavanols and Flavonols Are Inversely Associated with Incident Type 2 Diabetes in European Populations

## Online Supporting Material

**Files in this Data Supplement:**

- Online Supporting Material - Tables 1-3
