# Supplementary material for: Dietary Intakes of Individual Flavanols and Flavonols Are Inversely Associated with Incident Type 2 Diabetes in European Populations
Source: J Nutr. 2013 Dec 24;144(3):335–43. doi: 10.3945/jn.113.184945 (PMC3927546; doi:10.3945/jn.113.184945)
Supplement: Online Supporting Material [file jn.113.184945_nutrition184945SupplementaryData1.doc]

**Online Supporting Material**

**Supplementary table 1.** Spearman correlations among flavan-3-ol monomer intakes in the EPIC-InterAct subcohort (n=15,258).^1^

|  | **Epigallocatechin**  **3-gallate** | | **Epicatechin**  **3-gallate** | **Epigallocatechin** | | **Epicatechin** | **Catechin** | **Catechin**  **3-gallate** | **Gallocatechin** |
| --- | --- | --- | --- | --- | --- | --- | --- | --- | --- |
| Epigallocatechin 3-gallate | 1.00 |  | |  |  | |  |  |  |
| Epicatechin 3-gallate | 1.00 | 1.00 | |  |  | |  |  |  |
| Epigallocatechin | 1.00 | 0.99 | | 1.00 |  | |  |  |  |
| Epicatechin | 0.85 | 0.85 | | 0.86 | 1.00 | |  |  |  |
| Catechin | 0.51 | 0.50 | | 0.52 | 0.70 | | 1.00 |  |  |
| Catechin 3-gallate | 1.00 | 1.00 | | 1.00 | 0.84 | | 0.50 | 1.00 |  |
| Gallocatechin | 1.00 | 0.99 | | 1.00 | 0.85 | | 0.53 | 1.00 | 1.00 |

^1^All correlations were highly statistically significant (P<0.001)

**Online Supporting Material**

**Supplementary table 2.** Spearman correlations among proanthocyanidin intakes in the EPIC-InterAct subcohort (n=15,258).^1^

|  | **PA dimers** | **PA trimers** | **PA 4-6mers** | | **PA 7-10mers** | **PA polymers** |
| --- | --- | --- | --- | --- | --- | --- |
| PA dimers | 1.00 |  |  |  | |  |
| PA trimers | 0.66 | 1.00 |  |  | |  |
| PA 4-6mers | 0.74 | 0.87 | 1.00 |  | |  |
| PA 7-10mers | 0.73 | 0.85 | 0.99 | 1.00 | |  |
| PA polymers | 0.70 | 0.80 | 0.94 | 0.94 | | 1.00 |

^1^All correlations were highly statistically significant (P<0.001)

**Online Supporting Material**

**Supplementary table 3.** Spearman correlations among flavonols intakes in the EPIC-InterAct subcohort (n=15,258).^1^

|  | **Quercetin** | **Kaempferol** | **Myricetin** | **Isorhamnetin** |
| --- | --- | --- | --- | --- |
| Quercetin | 1.00 |  |  |  |
| Kaempferol | 0.63 | 1.00 |  |  |
| Myricetin | 0.74 | 0.68 | 1.00 |  |
| Isorhamnetin | 0.50 | 0.05 | 0.11 | 1.00 |

^1^All correlations were highly statistically significant (P<0.001)
